# Compositional changes and ecological characteristics of earthworm mucus under different electrical stimuli

**DOI:** 10.1038/s41598-023-29125-7

**Published:** 2023-02-09

**Authors:** Huihui Huan, Xingming Wang, Zhaoxia Chu, Xiaokun Yu, Tingyu Fan, Gang Li, Xiaoping Xu, Quan Zhen, Luntao Sun, Zhongbing Dong, Shijiao Zha

**Affiliations:** 1 Anhui Province Engineering Laboratory of Water and Soil Resources Comprehensive Utilization and Ecological Protection in High Groundwater Mining Area, School of Earth and Environment, Anhui University of Science and Technology, Huainan, 232001 China; 2 State Key Laboratory of Safety and Health for Metal Mines, Sinosteel Maanshan General Institute of Mining Research Company Limited, Maanshan, 243000 China; 3 Collaborative Innovation Center of Recovery and Reconstruction of Degraded Ecosystem in Wanjiang Basin Co-Founded By Anhui Province and Ministry of Education, School of Ecology and Environment, Anhui Normal University, Wuhu, 241002 China; 4 Engineering Laboratory of Comprehensive Utilization and Ecological Protection of Soil and Water Re-Sources in High Diving Level Mining Area of Anhui Province, Huainan, 232001 China; 5 Institute of Environment-Friendly Materials and Occupational Health, Anhui University of Science and Technology (Wuhu), Wuhu, 241000 China; 6 Key Laboratory of Bioresource and Environmental Biotechnology of Anhui Higher Education Institutes, School of Biological Engineering, Huainan Normal University, Huainan, 232038 China; 7 College of Civil Engineering and Architecture, Anhui Polytechnic University, Wuhu, 230009 China; 8 Department of Preventive Medicine, Bengbu Medical College, Bengbu, 233033 China

**Keywords:** Biochemistry, Psychology, Biogeochemistry, Ecology, Environmental sciences

## Abstract

Earthworm mucus is rich in nutrients that can initiate the mineralization and humification of organic matter and is of great importance for contaminated soil remediation and sludge reutilization. In this study, six voltage and current combinations were utilized to promote earthworm mucus production (5 V and 6 V at 10, 20 and 30 mA, respectively), to explore the compositional changes of the mucus produced under different electrical stimuli, and to propose the best electrical stimulation group and mucus fraction applicable to soil heavy metal pollution remediation and sludge reutilization. The results showed that the mucus produced by the six electrical stimuli was mainly composed of proteins, amino acids, carbohydrates, fatty acids, and polysaccharides, with small amounts of alcohol, phenol, and ester organic substances. Under different electrical stimuli, each component changed significantly (P < 0.05). pH and conductivity were higher at 6 V 20 mA, total nitrogen and phosphorus contents reached their maximum at 5 V 30 mA, and total potassium at 6 V 10 mA. Protein, amino acids, and carbohydrates were most abundant in the mucus produced at 5 V 10 mA, while trace metal elements reached their lowest values at 5 V 10 mA. Finally, based on principal component analysis and combined with previous studies, it was concluded that the mucus produced at 5 V 10 mA was weakly alkaline, high in amino acids and nutrients and low in trace metal elements, and most suitable for sludge and straw composting experiments, soil remediation and amendment experiments.

## Introduction

Earthworms are invertebrates that live in soil and often function as ecological engineers in soil ecosystems^1^. From a macroscopic perspective, earthworms promote the formation of soil aggregates through burrowing, foraging, moving, and casting and thus increase the level of soil mineralization, improve soil permeability, and enhance water retention capacity. Earthworm castings are also rich in humus and plant nutrients and a pollution-free, sustainable, and efficient organic fertilizer^2–4^. At the microscopic level, earthworms can change the richness and diversity of the microbial community, thereby accelerating the utilization of organic matter, and also have the ability to absorb and become enriched for heavy metals^5,6^. Earthworms and their surrounding environment are also collectively referred to as the “earthworm contact circle.” When earthworms are active in an “earthworm contact circle”, they release a light-yellow mucus with a pungent odor from their epidermis, which is a natural protective barrier of earthworms. Moreover, this mucus contains proteins, amino acids, carbohydrates, and other substances that are essential for earthworm growth, development, reproduction, and resistance^7–9^.

Studies have shown that earthworm mucus contains immune cells, antimicrobial peptides, antimicrobial proteins, and hemagglutination that act in a phagocytic role, provide resistance against bacteria, and protect earthworms from pathogens^10^; mucus also contains compounds such as insecticidal, antifungal, and antiviral proteins and phytohormones that inhibit the growth of plant pathogens, such as *Fusarium oxysporum* and *Candida albicans*, thereby improving seed germination and promoting plant growth potential^10,11^. Earthworm mucus is also particularly useful to soil and growing plants. The amino acids in mucus are an easily absorbed liquid fertilizer that provides abundant nutrients to plants, promotes the growth and Cd tolerance of tomato seedlings, increases the accumulation of Cd in plants, and also enhances microbial activity in the substrate, thus increasing microbial enrichment by 3.4–11 fold^4,5,12^. Bityutskii et al.^13^ found that mucus can drive and accelerate the level of mineralization and humification of plant residues, resulting in strong qualitative changes in humus composition. Similarly, Pan et al.^14^ found that mucus contains ligands that effectively complex organic contaminants, alter the distribution of organic contaminants in the soil, improve the bioavailability of contaminants, and promote bioremediation. Sizmur et al.^15^ also found that mucus had a significant effect on the transport and morphology of the heavy metal arsenic in contaminated soils.

Therefore, mucus can have important ecological roles in organic matter stabilization, microbial community succession, heavy metal regulation, seed germination, plant pest and disease prevalence, plant growth, and soil remediation. Currently, mucus extraction methods are complex and varied, including natural, distilled water, stirring, thermal stimulation, sterile water, quartz sand, and electrical stimulation methods, among which quartz sand^6^ and electrical stimulation methods^16^ are widely used in scientific experimental research because of their ease of extraction and high collection efficiency. However, the physicochemical properties of mucus may change among extraction methods^7,11,17^. Previously, Sizmur et al.^15^ used the quartz sand method to extract mucus produced by earthworms at different densities and found significant changes in the physicochemical properties of the mucus, but few detailed studies have reported the characteristics of changes in the mucus properties produced by stimulating earthworms with different voltages and currents. Therefore, the goal of the present research was to investigate the variation patterns of earthworm mucus properties under different electrical stimulation treatments, and to determine mucus components and electrical stimulation treatments groups applicable to soil heavy metal pollution remediation and sludge reutilization, so as to provide a basic data reference for subsequent ecological studies on the application of mucus to contaminated soil remediation and sludge resource recovery, for example.

In this experiment, based on the extraction of earthworm mucus without any blood or other liquid contamination after 5 V and 6 V electrical stimulation of earthworms by Kobayashi et al.^18^, Aja et al.^16^, and AIlegretta et al.^19^, the current was refined, and earthworm mucus was extracted after combined low voltage (5 V, 6 V) and low current (10, 20, 30 mA) methods were applied. We investigated (1) changes in physicochemical factors and nutrient elements of earthworm mucus under different electrical stimuli, (2) changes in organic functional groups and amino acid contents, (3) correlations between mucus levels of physicochemical factors, nutrient elements, and amino acids, (4) changes in trace element contents, (5) factor principal component analysis, and (6) the most appropriate research objectives for mucus production under different voltage and current settings.

## Materials and methods

### Earthworm mucus collection

All experimental procedures described in this study were performed in accordance with the Guide for the Care and Use of Laboratory Animals and complied with the ARRIVE guidelines. Earthworms (*Eisenia foetida*) were purchased from an earthworm breeding center in Jurong, Jiangsu province, China. The purchased earthworms were acclimated to the laboratory for 7 days and then subjected to mucus extraction. Adult earthworms with strong physical activity and obvious ring bands and weighing 0.35–0.5 g were selected, rinsed with distilled water, and placed in a defecation box for 24 h in the dark, after which they were rinsed and dried again. The earthworms were divided into six 300 g groups and electrically stimulated at 5 V (10, 20, 30 mA) and 6 V (10, 20, 30 mA) using an adjustable DC power supply to induce secretion of earthworm mucus. Three 60 s electrical stimulations separated by intervals of 60 s were performed on each of the six treatment groups. These operations were repeated for each treatment group three times, and a total of 5400 g of earthworms were used. The earthworms survived the electrical shocks and were returned to the laboratory earthworm incubator for further rearing. The amount of mucus obtained from each group that received electrical stimulation was about 25–30 g. The collected mucus was centrifuged at 5031 xg for 10 min to obtain pure earthworm mucus supernatant without cellular components, which was stored in a − 20 °C freezer for subsequent determination.

### Basal mucus indicators and amino acid determination

Both pH and electrical conductivity (EC) were determined by a benchtop acidity meter and benchtop conductivity meter using the method described by Nadana et al.^11^. Total nitrogen (TN) and total phosphorus (TP) were determined by the combined alkaline potassium persulfate method^20^, and Total potassium (TK) was determined by perchloric acid-hydrofluoric acid digestion^7^. Amino acid fractions were determined based on the methods described in the National Standard of the People's Republic of China for the Determination of Amino Acids in Food (GB 5009.124-2016)^21^ as follows. First, 5 mL of mucus was mixed with 5 g of 6 mol/L hydrochloric acid. Then, the tube was flushed with nitrogen for 15 min with a dry nitrogen blowing apparatus (LICHEN, LC-DCY-12G), sealed, and placed in an oven at 110 °C for 22–24 h. The resulting solution was cooled and volumed to 100 mL with ultrapure water. Then, 2 mL of the fixed solution was deacidified in the nitrogen blowing apparatus until dry and dissolved with 2 mL of sodium citrate buffer solution (pH d). The solution was dissolved, shaken, mixed, and then passed through a 0.22-μm filter column for the determination of aspartic acid (Asp), threonine (Thr), serine (Ser), glutamic acid (Glu), glycine (Gly), alanine (Ala), cysteine (Cys), valine (Val), methionine (Met), isoleucine (Ile), leucine (Leu), tyrosine (Tyr), phenylalanine (Phe), histidine (His), lysine (Lys), arginine (Arg) and proline (Pro) using a Hitachi Amino Acid Analyzer (Hitachi L-8900).

### Fourier transform infrared spectroscopy of mucus

Using the Fourier transform infrared spectroscopy (FTIR) method described by Aja et al.^16^, 5 g of earthworm mucus was freeze-dried in a vacuum freeze-dryer (LGJ-18S, Shanghai Yuming Instrument Equipment Co, LTD., Shanghai, China) at − 50 °C. After 48 h, the dried mucus samples were removed, mixed with potassium bromide of guaranteed reagent in an agate mortar and ground into uniform transparent flakes with a 100 MPa tablet press, and placed in a Fourier transform infrared spectrometer (Nicolet IS50, Thermo Fisher, Waltham, MA, USA) with the number of scans set to 65 and a spectral range of 400–4000 cm^−1^, and the data were recorded at a resolution of 4 cm^−1^. The water content of earthworm mucus was also determined by the freeze-drying method in a vacuum freeze-dryer (LGJ-18S, Shanghai Yuming Instrument Equipment Co, LTD., Shanghai, China)^14^. The mucus water content produced by 5 V 10 mA, 5 V 20 mA, 5 V 30 mA, 6 V 10 mA, 6 V 20 mA, and 6 V 30 mA electrical stimulation was 98.9%, 98.7%, 98.8%, 99.2%, 98.9% and 98.7%, respectively.

### Determination of trace metal elements in mucus by ICP-MS

Trace metal elements were analyzed using the method described by Chen et al.^22^. First, 0.5 mL of mucus and 8 mL of concentrated nitric acid were mixed in a crucible, slowly heated and evaporated to 1 mL on an electric hot plate with a gradient of 140 °C, 160 °C, and 180 °C, and then filtered through a microporous membrane after cooling. Afterwards, ultrapure water was added to the sample to bring the volume to 25 mL, and ICP-MS (NexION300X, PerkinElmer, Wellesley, USA) was used to assay the following trace elements: Al, Mg, Fe, Cu, Cr, Zn. Mn, Ni, Pb, and K. National standards were assayed for quality control during the analysis and testing, including duplicate samples and blank reagents, and the sample recoveries and relative standard deviations were less than 10%, which met the experimental requirements.

### Statistical analysis

Data processing was performed using Excel 2021 (Microsoft Corp., Redmond, WA, USA) and SPSS24 (IBM Corp., Armonk, NY, USA) for mean and standard deviation calculations. Single factor analysis of variance (ANOVA) and multiple comparisons (LSD) were used to analyze physicochemical factors, nutritional elements, and trace metal elements in mucus under different electrical stimulation conditions with significance determined at 0.05 and 0.01 levels, and Fourier transform infrared analysis, correlation analysis, principal component analysis, and conventional graphing were performed using ORIGIN2021 (OriginLab, Northampton, MA, USA).

## Results and discussion

### Differences in mucus physicochemical factors and nutrient elements among electrical stimuli

#### Physical and chemical factors

**Figure 1:**
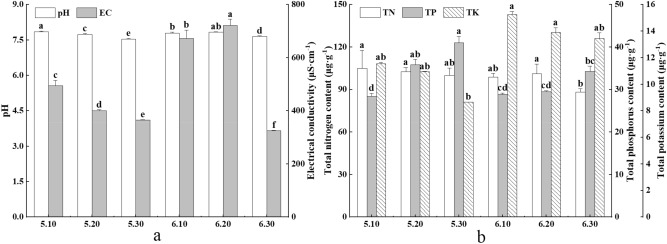
Changes in physicochemical factors and nutrient elements in earthworm mucus induced by different electrical stimuli. 5.10, 5.20, 5.30, 6.10, 6.20, 6.30 indicate 5 V 10 mA, 5 V 20 mA, 5 V 30 mA, 6 V 10 mA, 6 V 20 mA, and 6 V 30 mA, respectively; Data are presented as the mean ± SD for three duplicates, n = 3. To test for significance difference between different treatment groups, one-way analysis of variance (ANOVA) was used. Different lowercase letters indicate significant differences of the same index under different electrical stimuli (*P* < 0.05); *EC* electrical conductivity, *TN* total nitrogen, *TP* total phosphorus, *TK* total potassium.

Mucus contains electrolytes, such as potassium and multivalent calcium and magnesium ions, which participate in the osmoregulation of the earthworm body to maintain the metabolic balance of the organism^7,23^. When earthworms are subjected to different stimuli, the mucus composition changes^10^. As shown in Fig. 1a, earthworms produced mucus with significant (*P* < 0.05) differences in pH and EC among the six different electrical stimuli. The mucus pH consistently exhibited weak alkalinity (7.50 < pH < 8.00) and reached a maximum value of 7.85 at 5 V 10 mA. The mucus pH also showed a tendency to decrease to different degrees as voltage and current increased, and the decrease was significant for most stimuli (*P* < 0.05, except for 6 V 20 mA, which was not significant), and a minimum value of 7.54 occurred at 5 V 30 mA. However, the EC value of mucus induced by different electrical stimuli varied more. EC significantly differed between all groups (*P* < 0.05), and the variation at 6 V was more obvious than that at 5 V. Additionally, both the maximum and minimum values appeared at 6 V, and the EC values of 6 V 10 mA and 6 V 20 mA were 1.5–2 times higher than those of other treatments (5 V 10 mA, 5 V 20 mA, 5 V 30 mA, 6 V 30 mA). Thus, different electrical stimuli induced clearly different effects in mucus EC.

External stimulation of cells changes the distribution of substances and potential of the surface of the cell membrane, which affects membrane permeability and alters the pH^24,25^. As mucus is produced by the outermost columnar gland cells and somatic lumen cells in the earthworm epidermis^10^, both pH and EC changed when electrically stimulated at different intensities. When the intensity of electrical stimulation increased, the permeability of the cell membrane gradually increased, promoting the secretion of small intracellular organic acids, NH_4_^+^, and other acids, resulting in a gradual decrease in pH^12,26^. The change in membrane permeability also increased the release of inorganic salts, metabolic waste and minerals, and some minerals were also converted into soluble forms in the form of electrolytic ionization^27^, changing the EC of the mucus such that it increased with voltage, resulting in a higher EC at 6 V. The EC of mucus abruptly decreased at 6 V 30 mA. At this point, the stimulation probably did not destroy the cell membrane integrity, and the earthworm secreted mucus while reducing material efflux in order to respond to the stress of stronger electrical stimulation and maintain body fluid homeostasis.

#### Nutritional elements

Mucus contains nutrients that are required for plant growth, such as N, P and K, so it is important to study changes in nutrient contents of mucus produced under different electrical stimuli^17^. As shown in Fig. 1b, TN and TK contents were lowest at 6 V 30 m A and 5 V 30 mA, respectively, significantly lower levels than those of the other treatment groups (Note: The other treatment groups for total nitrogen were 5 V 10 mA, 5 V 20 mA, 5 V 30 mA, 6 V 10 mA, 6 V 20 mA, and total potassium was 5 V 10 mA, 5 V 20 mA, 6 V 10 mA, 6 V 20 mA, 6 V 30 mA; *P* < 0.05), and there was no significant difference between the remaining five groups (Note: The other treatment groups for total nitrogen were 5 V 10 mA, 5 V 20 mA, 5 V 30 mA, 6 V 10 mA, 6 V 20 mA, and total potassium was 5 V 10 mA, 5 V 20 mA, 6 V 10 mA, 6 V 20 mA, 6 V 30 mA; *P* > 0.05), with TN and TK higher at 5 V and 6 V, respectively. The variation in TP among electrical stimuli was greater, and the electrical stimuli treatments can be ranked in the following descending order of TP content: 5 V 30 mA, 5 V 20 mA, 6 V 30 mA, 6 V 20 mA, 6 V 10 mA, 5 V 10 mA. Although the lowest TP content appeared at 5 V, the content of TP was generally higher at 5 V voltage.

Mucus is relatively rich in N, P, and K. Mucus N is mainly derived from substances such as NH_4_^+^, urea, small molecule metabolites, amino acids, and proteins^26,28^. In contrast, mucus P is derived from bacterial microorganisms and organophosphorus compounds^7^, while mucus K is mainly in cytoplasm and electrolytes^29,30^. Under 5 V voltage stimulation, the earthworm body surface normally released mucus, which contains various nutrients required by plants. As the voltage increased to 6 V, the stimulation and pressure on the earthworm increased, inducing the release of more mucus, while the earthworm also released more potassium ions into the mucus in order to maintain the balance of sodium and potassium ions in its body fluid, resulting in a higher potassium ion content at 6 V^31^. While the cell membrane remained intact and active throughout the process, it was still selectively permeable to organic material and did not allow the excessive flow of organic material into the mucus^32^. Therefore, N and P from organic matter fractions were higher at 5 V, while inorganic salt ions K were higher at 6 V.

### FTIR and amino acid differences of mucus among electrical stimuli

**Figure 2:**
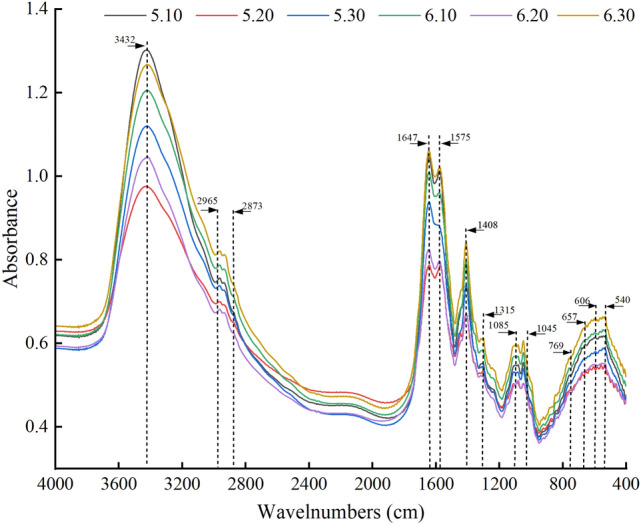
Fourier transform infrared spectroscopy (FTIR) functional group changes in earthworm mucus under different electrical stimuli. 5.10, 5.20, 5.30, 6.10, 6.20, 6.30 indicate 5 V 10 mA, 5 V 20 mA, 5 V 30 mA, 6 V 10 mA, 6 V 20 mA, and 6 V 30 mA, respectively.

The FTIR spectra can reflect the chemical structure and functional groups of organic substances; thus, the infrared spectra of earthworm mucus produced under different voltage–current combinations show that the absorption peaks of all groups of mucus appeared at 3432 cm^−1^, 2965–2873 cm^−1^, 1647 cm^−1^, 1575 cm^−1^, 1408 cm^−1^, 1315 cm^−1^_,_ 1085–1045 cm^−1^, and 769–540 cm^−1^ (Fig. 2). Additionally, among treatments, the waveforms and crests were very similar, while the intensity of the crests changed significantly. Absorption peaks at 3432 cm^−1^ correspond to carbohydrates and proteins, while those at 2965–2873 cm^−1^ correspond to fatty acids mainly subjected to anti-symmetric stretching vibrations of C–H_3_ and C–H. Peaks at 1647 cm^−1^ correspond to C = O stretching binding, N–H bending and C-H variable angle vibrations of amide I bands and polysaccharide absorption peaks, while the peak appearing at 1575 cm^−1^ is probably the amide II bands of C–N stretching and N–H bending. The peak at 1408 cm^−1^ can be attributed to C–O asymmetric stretching, O–H bending vibrations, and symmetric stretching of COO– from carboxyl groups, while C–O stretching vibrations and O–H planar bending vibrations of ethers, phenols and esters would appear at 1315 cm^−1^. The broad peak at 1085–1045 cm^−1^ mainly corresponds to C–O–C and C–H–O functional groups of alcohols and polysaccharides and H–C–H stretching vibrations, and O–C–N bending, out-of-plane C = O bending of amide IV and out-of-plane N–H bending of amide V appear at 769–540 cm^−1^^7,16,33,34^. It is thus clear that earthworm mucus in this experiment consisted mainly of proteins, carbohydrates, fatty acids, and polysaccharides and contains small amounts of organic substances such as alcohols, phenols and esters. Thus, the absorption spectra of proteins and carbohydrates dominate substantially, which is also consistent with the results of Guhra et al.^7^.

Of the six bands in the spectral range of 400–4000 cm^−1^, 3432 cm^−1^, 1647 cm^−1^, and 1575 cm^−1^ were the three most dominant absorption bands, representing water-soluble proteins and amide I and II bands, respectively. Additionally, 9 of the 20 amino acids that constitute proteins showed strong absorbance corresponding to the amide I and II bands, so the amide I and II bands could also reflect the amino acid content^16,35^. The absorbance of 5 V 10 mA, 6 V 10 mA, and 6 V 30 mA in this band was at a higher level in the Fig. 2, indicating high protein and amino acid content, followed by 5 V 20 mA in the middle, and finally 5 V 20 mA and 6 V 20 mA with the lowest absorption wavelengths. Thus, it was found that the protein and amino acid contents of the mucus produced under different electrical stimuli changed significantly, probably owing to the presence of defense proteins in the earthworm, which secrete amino acids with immune functions to respond to acute stress after different stimuli^36,37^.

To further explain the mechanism of protein amino acid changes in mucus under different electrical stimuli, proteins in mucus were hydrolyzed, and the changes in the total content of 17 amino acids in mucus were analyzed^12^. As shown in Table 1, glutamic acid, alanine, aspartic acid, proline, and leucine were the most dominant components of the observed 17 amino acids (44.6–47.4%), with glutamic acid being the most abundant at 13.2–14.3%, while cystine accounted for only 0.3–0.4% of all amino acids and was the least abundant in the mucus. Under the six electrical stimuli, the vast majority of amino acids reached the highest and lowest levels in the mucus at 5 V 10 mA and 5 V 20 mA, respectively, with total concentrations of 4.3 and 3.6 mg/g, while the amino acid content in the 6 V treatment groups changed more moderately, without any large fluctuations at 5 V. The stimuli can be ranked in descending order of their amino acid content as follows: 5 V 10 mA, 6 V 30 mA, 6 V 10 mA, 6 V 20 mA, 5 V 30 mA, 5 V 20 mA. Among all amino acids, except alanine, cystine, and arginine, the amino acid content of 5 V 10 mA mucus was higher than the amino acid content of other treatment groups. Among them, the content of glycine, phenylalanine, threonine, proline, and tyrosine at 5 V 20 mA decreased at a higher rate compared to 5 V 10 mA, by 32.4%, 28.4%, 28.0%, 26.4% and 26.1%, respectively. In comparison, alanine and cystine reached a maximum at 5 V 30 mA, with an elevation of 13.8% and 16.0% compared to 5 V 10 mA, and arginine at 6 V 30 mA reached a maximum value, which was 8.4% higher than 5 V 10 mA.

**Table 1:** Changes in amino acid content in earthworm mucus under different electrical stimuli.

Amino acid	5.10	5.20	5.30	6.10	6.20	6.30
	Content (mg·g^−1^)					
Asp	0.356	0.308	0.334	0.351	0.338	0.338
Thr	0.214	0.154	0.175	0.194	0.192	0.175
Ser	0.184	0.146	0.161	0.175	0.165	0.168
Glu	0.578	0.522	00563	0.545	0.547	0.568
Gly	0.225	0.152	0.170	0.193	0.187	0.173
Ala	0.365	0.387	0.416	0.292	0.311	0.410
Cys	0.014	0.011	0.016	0.016	0.015	0.013
Val	0.268	0.208	0.241	0.250	0.239	0.225
Met	0.138	0.138	0.123	0.133	0.134	0.134
Ile	0.241	0.205	0.235	0.235	0.232	0.216
Leu	0.318	0.268	0.295	0.295	0.301	0.296
Tyr	0.195	0.144	0.167	0.167	0.173	0.184
Phe	0.273	0.196	0.253	0.253	0.241	0.252
His	0.172	0.151	0.157	0.157	0.160	0.167
Lys	0.316	0.261	0.284	0.284	0.278	0.285
Arg	0.179	0.159	0.173	0.173	0.168	0.194
Pro	0.339	0.249	0.265	0.312	0.300	0.271
Total	4.376	3.660	3.976	4.026	3.981	4.069

5.10, 5.20, 5.30, 6.10, 6.20, 6.30 indicate 5 V 10 mA, 5 V 20 mA, 5 V 30 mA, 6 V 10 mA, 6 V 20 mA, and 6 V 30 mA, respectively.

Among the nine amino acids that correspond to the amide I and II bands, this mucus contains seven, namely glutamic acid, aspartic acid, lysine, phenylalanine, tyrosine, arginine, and histidine^16^, accounting for 47.3% to 48.8% of the total amino acid content, which is also consistent with Fig. 2. Glutamic acid is the most abundant excitatory neurotransmitter in the central nervous system and can stabilize the central nervous system^38^, and aspartic acid performs important roles related to nervous system development and hormone regulation^39^. Additionally, lysine has a stress-protective function against external stimuli^40^. Accordingly, the content of these three amino acids is high in the mucus. Earthworm mucus also exhibited changes in amino acid fractions under stimulation. For example, exposure of earthworms to Cu produced histidine^41^, which had a detoxification mechanism, and exposure to pesticides causes significant increases in isoleucine, alanine, and glutamate^42^, which can also be used as indicators in earthworm exposure tests^43^. Thus, when earthworms are electrically stimulated, they protect themselves by changing the humoral environment through stress responses. For example, aspartic acid has a protective effect on the myocardium, and electrical stimulation can change its secretion to maintain the electrolyte balance of the animal’s myocardium and thus myocardial function^39^. Additionally, glutamate affects the central nervous system of earthworms under electrical stimulation, and its secretion was indeed changed. Earthworms also produce defense proteins to protect themselves from damage, and there are also 16 amino acids present in the five earthworm defense proteins identified by Roch et al.^44^; these defense proteins are also present in the mucus to protect the earthworm. It is thus clear that earthworms produce different amino acids to regulate homeostasis in the body when subjected to different electrical stimuli, allowing the earthworm to protect itself from damage in a cost-effective manner. Among the six voltage–current combinations examined, 5 V 10 mA stimulated the earthworms the least and induced the most abundant levels of amino acids.

### Correlation between levels of physicochemical factors, nutritional elements, and amino acids in mucus under different electrical stimuli

**Figure 3:**
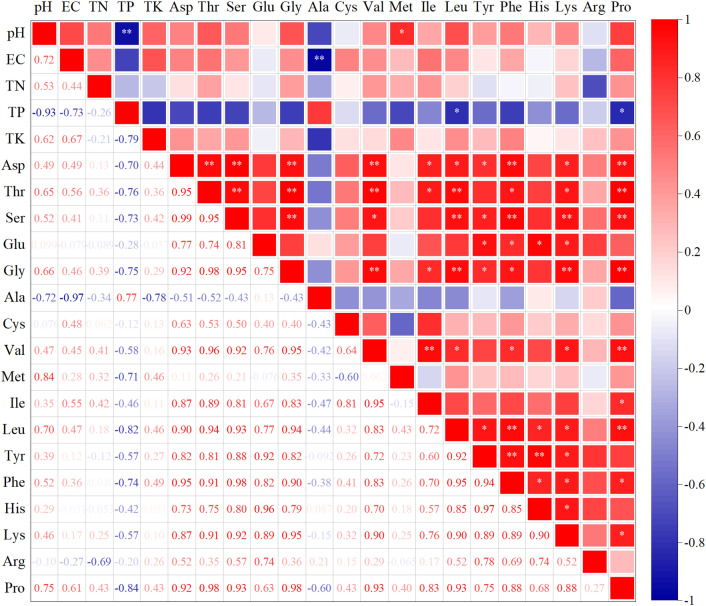
Correlations between the contents of substances in earthworm mucus under different electrical stimuli. Red and blue indicate significant positive and negative correlations, respectively; the darker the red or blue color, the greater the absolute value of the correlation coefficient; ** means correlation is significant at the 0.01 level, * means correlation is significant at the 0.05 level.

The physiochemical factors, nutrient elements, and amino acids in mucusre crucial for plant growth, organic matter mineralization, soil remediation and sludge compost stabilization and are among the current criteria for evaluating the performance of mucus. Therefore, it is important to conduct correlation analysis to explore the intrinsic connection between different voltage currents^5,7,12,16^. As shown in Fig. 3, there were significant negative correlations of pH with TP (*P* < 0.05), EC with Ala (*P* < 0.01), TP with Leu and Pro (*P* < 0.05), and pH with Met (*P* < 0.05) at various voltage and current combinations, while the correlations between levels of amino acids varied more closely. The contents of the 10 amino acids Asp, Thr, Ser, Gly, Val, Ile, Leu, Phe, Lys, and Pro all showed significant positive correlations with each other (*P* ≤ 0.05, except Ile, Ser, Leu, Phe, Lys), and levels of Glu, Tyr, and His also showed significant positive correlations with each other (*P* ≤ 0.05). However, levels of Ala, Cys, Met, and Arg were not significantly correlated with each other or with those of other amino acids (*P* > 0.05). Indeed, positive correlations between levels of amino acids are likely to indicate common origins^45^. Based on the data in Table 1, it was further found that Asp, Thr, Ser, Gly, Val, Ile, Leu, Phe, Lys, and Pro showed the same trend, and Glu, Tyr, and His showed almost the same trend. Thus, Asp, Thr, Ser, Gly, Val, Ile, Leu, Phe, Lys, and Pro appear to have the same origin in the production of mucus at various voltage–current combinations, and Glu, Tyr, and His similarly originated from the same source conditions.

### Differences in contents of trace elements in mucus among electrical stimuli

**Figure 4:**
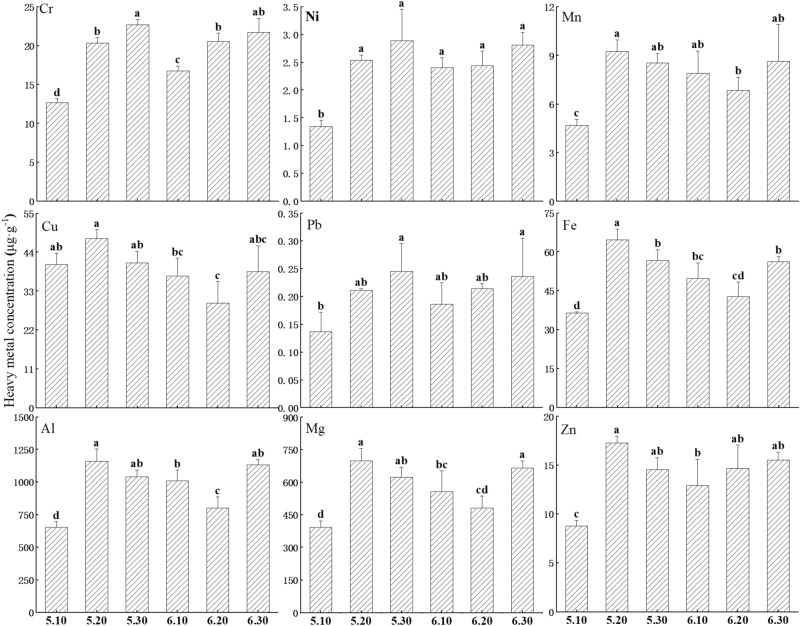
Differences in trace elements in earthworm mucus among electrical stimuli. 5.10, 5.20, 5.30, 6.10, 6.20, 6.30 indicate 5 V 10 mA, 5 V 20 mA, 5 V 30 mA, 6 V 10 mA, 6 V 20 mA, and 6 V 30 mA, respectively. Data are presented as the mean ± SD for three duplicates, n = 3. To test for significance difference between different treatment groups, one-way analysis of variance (ANOVA) was used. Different lowercase letters indicate significant differences of the same index under different electrical stimuli (*P* < 0.05).

As shown in Fig. 4, the following nine trace metal elements were identified in the mucus produced under different electrical stimuli in the following descending order of content: Al, Mg, Fe, Cu, Cr, Zn, Mn, Ni, and Pb. Among these trace elements, their contents were 652.6–1159.8 μg/g, 392.4–697.8 μg/g, 36.4–64.7 μg/g, 29.7–47.9 μg/g, 12.7–22.7 μg/g, 8.8–17.3 μg/g, 4.7–9.3 μg/g, 1.3–2.9 μg/g, and 0.14–0.25 μg/g respectively. The Al and Mg content is much higher than the other metal elements. In the mucus produced by earthworms under different electrical stimuli, the lowest content of all metallic elements occurred at 5 V 10 mA, except for Cu at 6 V 20 mA. Among them, the contents of Al, Mg, Fe, Cr, Zn, Mn, Ni, and Pb were significantly decreased at 5 V 10 mA compared to other treatment groups by 22.6–77.7%, 22.5–77.8%, 17.3–77.8%, 32.3–79.0%, 46.9–96.7%, 46.1–97.1%, 78.1–114.4%, and 36.4–79.4% (Note: The other treatment groups were 5 V 20 mA, 5 V 30 mA, 6 V 10 mA, 6 V 20 mA, 6 V 30 mA, respectively, *P* < 0.05). Moreover, the Cu content at 6 V 30 mA was significantly lower than the other treatment groups, 25.5%-61.2% (Note: The other treatment groups were 5 V 10 mA, 5 V 20 mA, 5 V 30 mA, 6 V 10 mA, 6 V 30 mA, respectively, *P* < 0.05). The analysis of the trends of the nine metal elements revealed that differences in Al, Mg, Fe, Cu, Zn, and Mn contents among the different voltage–current combinations were almost the same, and their contents could all be ranked in the same descending order according to treatment: 5 V 20 mA, 6 V 30 mA, 5 V 30 mA, 6 V 10 mA, 6 V 20 mA, 5 V 10 mA (except Cu at 5 V 10 mA and Zn at 6 V 20 mA). There were significant differences in trace metal levels between these treatments (*P* < 0.05), while Cr, Ni, and Pb showed similar trends in their change, but non-significant differences (*P* < 0.05) between multiple electrical stimuli treatments and thus less variability in metal element level.

Earthworms, as ecological engineers in soil ecosystems, can bioaccumulate and utilize different metal elements, and thus, their body metal element content also depends on their environment^46^. For example, Song et al.^47^ found that the Cu, Zn, and Pb contents of earthworms living in a mixed substrate of pig manure and mushroom residues increased significantly, while Zhang et al.^48^ found that earthworms living in soil with high levels of Cu, Cd, Pb, and Zn contamination also had elevated metal contents in their bodies. In contrast, earthworms in the present experiment lived in a mixed substrate of fermented cow dung and mud, and as the earthworms grew and reproduced, the metal elements in the substrate continuously accumulated in their bodies, thus affecting the metal element content in the mucus. The contents of Al and Mg were higher, probably owing to the substrate, and possibly because Al and Mg have important biological roles^49^. After earthworms received different electrical stimuli, the content of each metal element in the mucus produced changed to some extent, probably because the transport mechanisms of metal elements inside and outside the cell changed, such as the membrane potential of the cell membrane surface, the osmotic pressure inside and outside cells, and transport proteins themselves^49^. At the same time cells also have sensors that regulate the storage of metal ions in order to differentiate them, and thus, the regulation of metal ions changed when the electrical stimulation changed^50^, which altered the release of metal ions as well.

### Principal component analysis of each factor under different voltage and current combinations

**Figure 5:**
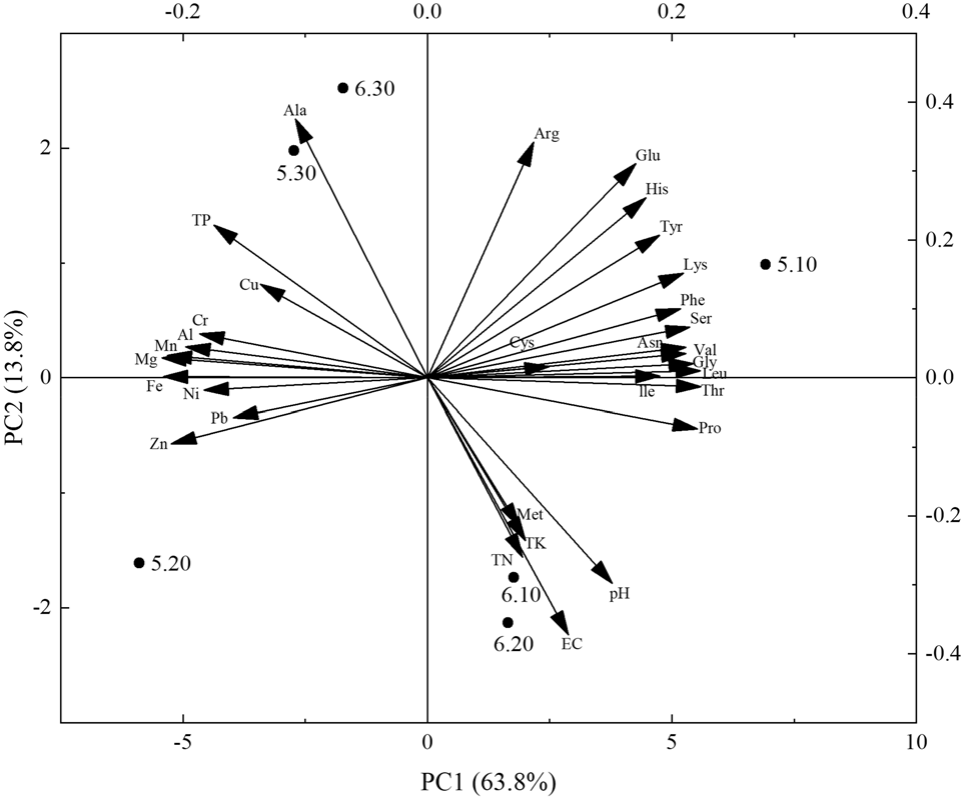
Principal component analysis of each factor in earthworm mucus induced by different electrical stimuli. 5.10, 5.20, 5.30, 6.10, 6.20, 6.30 indicate 5 V 10 mA, 5 V 20 mA, 5 V 30 mA, 6 V 10 mA, 6 V 20 mA, and 6 V 30 mA, respectively; *EC* electrical conductivity, *TN* total nitrogen, *TP* total phosphorus, *TK* total potassium.

To further clarify the effects of different voltage and current combinations on the physicochemical factors, nutrients, amino acids, and trace elements in the mucus produced, principal component analysis (PCA) was conducted (Fig. 5). Based on the PCA, a cumulative variance contribution of 77.57% for the two principal components was observed, with the first and second principal components (PC1 and PC 2 respectively) accounting for 63.8% and 13.8% of the variance. As shown in Fig. 5, 5 V 10 mA and 6 V 30 mA had significant positive effects on each factor in the PC1 and PC2 directions, respectively, and 5 V 20 mA and 6 V 20 mA had significant negative effects on each factor in the PC1 and PC2 directions, respectively; thus, it is clear that both 5 V 10 mA and 6 V 30 mA and both 5 V 20 mA and 6 V 20 mA are the main electrical stimulation groups affecting high and low levels of various factors inn mucus fractions, respectively. In addition, the factor loading of the two main components of each factor was determined to further clarify the contribution of each factor to the two main components, revealing that metal elements and most amino acids had the most significant negative and positive effects on PC1, respectively. Similarly, the factors pH, EC, TK, TP, and TK and the factors Glu, Ala, Tyr, His, and Arg had the most significant negative and positive effects on PC2. Figure 5 also shows three aggregations of mucus fractions produced by different electrical stimuli. Thus, pH, EC, TK, TK, and Met were more densely aggregated into one cluster, trace elements and TP were aggregated into another cluster, and the majority of amino acids were aggregated into the third cluster. This shows that the mucus extracted after stimulation with 5 V 10 mA was rich in amino acids and low in metal elements, the mucus extracted after stimulation with 5 V 20 mA was rich in metal elements and low in amino acids, while the mucus extracted after stimulation with 6 V 20 mA was richer in physicochemical factors and nutrient elements, with higher average levels of amino acids and metal elements.

### Evaluation of appropriate applications for mucus generation under different electrical stimuli

From the above analysis, it is clear that the physicochemical factors, nutrient elements, amino acids, and trace elements in earthworm mucus induced by six different voltage–current combinations significantly differed; therefore, the object of study for earthworm mucus application may change with the composition. As shown in Table 2, which is in part based on previous studies on earthworm mucus, it is clear that mucus that can have an important ecological impact on soil, compost, and plants should have the following specific characteristics: neutral pH, low EC, and exogenous amino acid protease content to promote the germination of plant seeds, high amino acid protein content to promote plant growth as a liquid fertilizer, the ability to adsorb clay minerals and complex to heavy metals. High contents of nutrients can not only promote plant growth, but also promote soil mineralization and humification, while also passivating heavy metals. The micronutrient content of mucus obtained is in the plant-available range, which can better replenish the micronutrients required by plants and has a bridging effect on the organic matter-mineral combination in the substrate.

**Table 2:** Properties and ecological functions of earthworm mucus.

Earthworm species	Mucus properties	Soil/compost/seed/plant changes	Authors
*Eudrilus eugeniae*	pH of 7.34, electrical conductivity of 250 us·cm^-1^, nitrogen content of 0.099%, and amino acid protease content	Induce seed germination at an earlier time and production of phenolic and antioxidant compounds, with up to 100% seed germination and high levels of shoot growth, root length, seedling growth and vigor	^17,51^
*Metaphire guiIlemi*	Rich in N, P, and K nutrients, various amino acids, and trace elements required by plants	Mucus provides sufficient nutrients and promotes faster growth of tomato seedlings, and the richness of amino acids in the mucilage has the potential to chelate metal ions, thus reducing the toxic and stressful effects of metals on plant growth, while also promoting the uptake and transport of essential elements by plants	^6,12,52^
*Aporrectodea caliginosa*	Rich in low-molecular-weight carbohydrates, ammonia, dissolved amino acids, and amino sugars	Priming effect on mineralization of plant residues and also positive effects on the quantity and quality of humus	^13,53^
*Lumbricus terrestris* and *Aporrectodea caliginosa*	High protein amino acid content and rich in both P and electrolytes (e.g., K^+^ and multivalent cations such as Mg^2+^, Al^3+^, Fe^3+^)	Mucus with high protein content has better adsorption of clay minerals, P has complete adsorption of iron ions, while abundant cations also promote the formation of organic mineral assemblages and improve the structure of soil aggregates through bridging effects	^7^
*Eisenia fetida*	Higher concentrations of ammonium, carbohydrates, dissolved nitrogen, phosphorus, and amino acids in the mucus	Mucus significantly accelerated the mineralization and humification of organic matter in the compost substrate, producing more dissolved carbon and significantly increasing the production of humus and fulvic acid-like substances. Mucus also increases the microbial abundance in the compost substrate, which reduces the effectiveness of heavy metals to some extent. The abundant amino acids also complex and adsorb with heavy metals, thus reducing the toxicity of heavy metals in sludge and increasing the quality of compost	^5,28,54^

Based on the present study, we can tentatively conclude and propose that the mucus produced under different electrical stimuli can be applied to different study subjects. Stimuli of 5 V 10 mA, 6 V 10 mA, and 6 V 20 mA can generate mucus to be used for sludge and straw composting tests, soil remediation and amendment tests, and the mineralization and humification of organic residues owing to its high amino acid content, high nutrient content, and low trace element content. Under the same conditions, 5 V 10 mA mucus with the highest amino acid content and the lowest trace elements was selected as the experimental group. The mucus induced by 5 V 30 mA stimulation had the most neutral pH, lower potassium ion content and EC, higher levels of amino acids, and moderate levels of trace elements, indicating it can be used for seed germination tests. The mucus induced by 6 V 30 mA had high contents of nutrients, amino acids, and trace elements essential for plants, making it particularly suitable for plant growth and development tests. Unlike mucus stimulated by 5 V 20 mA, which contains lower levels of amino acids but was richest in nutrients and trace elements, it can be used for fertilizing plants deprived of trace elements and for improving soil agglomerate structure tests.

## Conclusion

The earthworm mucus induced by different electrical stimuli all contained organic substances such as amino acids, proteins, fatty acids, polysaccharides, alcohols, phenols and esters as well as various trace elements. In the mucus produced under six different voltage and current combinations, changes in pH, EC, TN, TP, TK, amino acids, and trace elements occurred likely owing to differences in cellular osmotic pressure, membrane potential changes, sodium and potassium pumps, and stress protection responses of the organism. The correlation analysis of physicochemical properties and amino acid contents also revealed that Asp, Thr, Ser, Gly, Val, Ile, Leu, Phe, Lys, and Pro had the same source in the mucus, as they varied together under different electrical stimuli, while Glu, Tyr, and His similarly originated from the same source mechanism. Finally, it was tentatively concluded based on PCA and previous studies that the mucus produced at 5 V 10 mA was suitable for sludge and straw composting experiments, soil remediation and amendment experiments, and mineralization and humification of organic residues, while the mucus induced at 5 V 20 mA was deemed suitable for micronutrient-deprived plant fertilization and soil aggregate structure experiments. Additionally, 5 V 30 mA mucus was determined to be suitable for seed germination experiments, and 6 V 30 mA mucus was determined to be suitable for plant growth and development tests.

## Data Availability

The datasets used and analyzed during the current study are available from the corresponding author on reasonable request.
